# Stem Cell Ophthalmology Treatment Study (SCOTS): Bone Marrow-Derived Stem Cells in the Treatment of Age-Related Macular Degeneration

**DOI:** 10.3390/medicines7040016

**Published:** 2020-03-28

**Authors:** Jeffrey N. Weiss, Steven Levy

**Affiliations:** 1 The Healing Institute, 1308 North State Road #7, Suite A/B, Margate, FL 33063, USA; jweissmd@aol.com; 2 MD Stem Cells, 3 Sylvan Road South, Westport, CT 06880, USA

**Keywords:** macula, degeneration, retina, stem cells, bone marrow stem cells, AMD, age-related macular degeneration

## Abstract

**Background:** Dry age-related macular degeneration (AMD) is one of the leading causes of vision loss in older patients. The macula accumulates drusen with loss of retinal pigment epithelial cells and photoreceptors. Abnormal subretinal neovascularization is absent. There is no effective drug therapy for dry AMD and a large proportion of patients progress to legal blindness from macular atrophy. The Stem Cell Ophthalmology Treatment Study (SCOTS) was conducted to assess the effect of bone marrow-derived stem cells (BMSCs) on dry AMD and other retinal and optic nerve diseases. **Methods:** Thirty-two eyes were treated with BMSC per the protocols in SCOTS. Provision of BMSCs in Arm 1 was via retrobulbar (RB), sub-tenons (ST) and intravenous (IV); Arm 2 via intravitreal, RB, ST and IV; Arm 3 via subretinal and IV. Patient age averaged 78 years old and ranged from 69 to 90. Visual acuity preoperatively ranged from counting fingers to 20/50-2 with an average preoperative LogMAR of 1.125. **Results:** Following treatment, 20 of 32 (63%) of eyes experienced improvement in visual acuity averaging 27.6% on LogMAR and ranging from 2.5% to 44.6%. The mean improvement in LogMAR was 0.963 with a standard deviation (SD) of 0.42. The visual acuity remained stable in 34% of treated eyes. One eye continued to worsen as a consequence of disease progression. The results showed high statistical significance with *p* ≤ 0.001. The procedures were conducted safely, and no complications were observed. **Conclusions:** Treatment of dry AMD with BMSC using the protocols developed in the SCOTS clinical trial has shown statistically significant clinical benefit improving visual acuity and potentially delaying visual loss in the disease.

## 1. Introduction

The Stem Cell Ophthalmology Treatment Study (SCOTS) is an institutional review board (IRB) registered and monitored human clinical study using autologous stem cells derived from the patient’s own bone marrow. The study is listed on the National Institutes of Health clinical registry website under NCT number 01920867. It is evaluating the treatment of various retinal and optic nerve diseases that typically have no existing effective approaches to address vision loss or progression. All patients entering the study receive active treatment—the history of the disease and the current status is used as control. The bone marrow is obtained from the posterior pelvis bilaterally and then processed to provide a stem cell concentrate referred to as bone marrow-derived stem cells (BMSCs). This concentrate is then used for treatment of the patient.

The scientific literature regarding the use of BMSCs has been supportive of their value in the potential treatment of dry AMD. Harris et al. reported that CD133 progenitor cells from bone marrow were capable of retinal pigment epithelium (RPE) repair by homing to the injured tissue, differentiating into RPE-like cells and allowing the recovery of visual cycling on electroretinogram (ERG) [1]. Gong reported subretinal injection of bone marrow mesenchymal stem cells (BM-MSCs) with their transdifferentiation into glial, photoreceptor and retinal pigment epithelial cells [2]. Jiang et al. showed a reduction in the size of laser retinal injuries following intravenous injection of BM-MSCs in mice, likely by reducing retinal cell apoptosis [3]. In reviewing the potential of stem cells in retinal regeneration, Becker et al. of the University College of London and Moorfields discuss multiple different stem cells including human Müller stem cells [4]. Further expanding on this area, Pesaresi et al. showed that endogenous BMSCs introduced into a murine retina fused with Müller cells and differentiated into ganglion and amacrine cells [5].

Our use of autologous BMSC in SCOTS is compliant with the United States Food and Drug Administration (FDA) utilization guidelines for autologous cellular material. When performed in such a compliant fashion, the FDA considers the use of autologous BMSCs as a “practice of medicine” and not an investigative new drug (IND). As SCOTS is performed in a compliant fashion, and this is not an IND, the FDA does not take a position to approve the procedure or not. 

The SCOTS study has no grants or other financial support. SCOTS is a patient-funded study and patients pay in order to participate. 

The protocol for the treatment of the original SCOTS is continued in the follow-on Stem Cell Ophthalmology Treatment Study II (SCOTS 2) which is also IRB registered and approved; NCT 03011541. Based on the experience gained and data collected from the initial SCOTS trial, it is now typical for patients in SCOTS 2 to be assigned to Arm 1, which provides retrobulbar, sub-tenons and intravenous injections of BMSC, avoiding intraocular procedures. 

In the original SCOTS trial, there was a much lower than anticipated incidence of adverse events (AE) such as retinal detachment following treatment with Arm 2 or 3. As we could detect no apparent difference in responses in patients treated in Arm 2 or 3 when compared to Arm 1, it was decided in the SCOTS 2 trial to enroll patients only in Arm 1 to reduce the potential incidence of AE to zero if possible.

## 2. Materials and Methods

The inclusion and exclusion patient criteria for SCOTS have been presented repeatedly in a number of prior published papers [6,7,8,9,10]. They are reiterated here to facilitate the readers’ access:

- Have objective, documented damage to the retina or optic nerve unlikely to improve OR have objective, documented damage to the retina or optic nerve that is progressive AND have less than or equal to 20/40 best-corrected central visual acuity in one or both eyes AND/OR an abnormal visual field in one or both eyes;
- Be at least 3 months post-surgical treatment intended to treat any ophthalmologic disease and be stable;
- If under current medical therapy (pharmacologic treatment) for a retinal or optic nerve disease be considered stable on that treatment and unlikely to have visual function improvement (for example, glaucoma with intraocular pressure stable on topical medications but visual field damage);
- Have the potential for improvement with BMSC treatment and be at minimal risk of any potential harm from the procedure;
- Be 18 years of age or older;
- Be medically stable and able to be medically cleared by their primary care physician or a licensed primary care practitioner for the procedure. Medical clearance means that in the estimation of the primary care practitioner, the patient can reasonably be expected to undergo the procedure without significant medical risk to health.

Exclusion criteria include:

- Patients who are not capable of an adequate ophthalmologic examination or evaluation to document the pathology;
- Patients who are not capable or not willing to undergo follow up eye exams with the Principal Investigator or their ophthalmologist or optometrist as outlined in the protocol;
- Patients who are not capable of providing informed consent;
- Patients who may be at significant risk to general health or to the eyes and visual function should they undergo the procedure.

There are three arms of SCOTS with the type of treatment chosen based on the degree of visual loss, etiology of visual loss, associated risk factors for the treatment arms and the patient’s medical risk status. Bilateral treatment is provided assuming both eyes meet eligibility requirements. As these are autologous stem cells, no immunosuppression is required. 

An FDA cleared Class 2 medical device is used to separate the bone marrow aspirate into a stem cell concentrate. This concentrate has averaged 1.2 billion total nucleated cells (TNCs) including mesenchymal stem cells in approximately 14–15 cm^3^ of concentrate. The concentrate was analyzed from a 1 cm^3^ sample via flow cytometry. No evaluation of specific Cluster Differentiation (CD) markers was performed. The retrobulbar injection consists of 3 cm^3^ of concentrate with an average of approximately 240 million TNCs; sub-tenons injection of 1 cm^3^ with an average of approximately 80 million TNCs; intravitreal injection of 0.05 cm^3^ with an average of approximately 4 million TNCs subretinal injection of approximately 0.1 cm^3^ with approximately 8 million TNCs and intra-optic nerve injection of approximately 0.1 cm^3^ with approximately 8 million TNCs. The remainder of the concentrate is injected intravenously. 

Arm 1 consists of the stem cell concentrate injected retrobulbar and sub-tenons followed by intravenous infusion. Patients with ophthalmic conditions that preclude safe or effective utilization of intravitreal injection of concentrate, such as the presence of silicone oil, may be offered Arm 1 if meeting inclusion criteria. 

Arm 2 consists of the administration of retrobulbar, sub-tenons and intravitreal concentrate followed by intravenous infusion. Patients meeting inclusion criteria with a visual acuity between 20/40 and 20/200 in one or both eyes and/or visual field loss may be offered Arm 2. 

Arm 3 is reserved for retinal and optic nerve patients with severe visual loss (visual acuity of 20/200 or worse in at least one eye). Typically, patients admitted to Arm 3 have poorer vision (less than 20/200). Arm 3 consists of the better-seeing eye receiving the same treatment as Arm 1 or more typically, Arm 2, and the eye with more extensive visual acuity loss receiving a core pars plana vitrectomy with injection of subretinal or intra-optic nerve concentrate followed by the infusion of intravenous stem cells. Monocular patients are not eligible for Arm 3. 

Follow up examinations are required at 1, 3, 6, and 12 months post-treatment with reporting of the eye exam results to the Principal Investigator and the Study Director. 

The study and data accumulation were carried out with approval from the National Institutes of Health (NIH) and Office for Human Research Protections (OHPR) compliant Institutional Review Board (IRB). Informed consent for the research was obtained from each participating patient, and the data is protected in accordance with HIPAA regulations.

The preoperative exclusion criteria were: no light perception (NLP) vision, presence of concurrent untreated or unstable ocular disease, patient unwillingness to sign the informed consent form, inability to adhere to prescribed behavior including avoidance of smoking, unwillingness to adhere to post-operative instructions, or obtain and provide required follow up eye exams and data. 

All patients enrolled in this study underwent a comprehensive ophthalmologic examination, including significant past medical and ocular history, best-corrected Snellen and Early Treatment Diabetic Retinopathy Study (ETDRS) visual acuities, anterior segment biomicroscopy, measurement of intraocular pressure and dilated fundus examination. Automated perimetry (if possible dependent on the degree of visual loss), ocular coherence tomography (OCT) and fundus photography were performed. If there was a suspicion of neovascularization, a fluorescein angiogram was done. The approximate duration of visual loss was obtained from the patient and from a review of the patient’s medical records. 

The visual acuity for patients with less than 20/400 on the Snellen chart (i.e., inability to see the largest projected Snellen optotype) was measured using a 20/200 “E” card held at varying premeasured distances until the patient reported visualization (the optotype was held in front of the patient but eccentric gaze was permitted). In reporting visions in eyes less than 20/1000, count fingers was recorded. During follow-up examinations, practitioners without a 20/200 “E” card were permitted to directly report count fingers (CF). Vision was judged to be hand motions (HM) if the hand-held optotype could not be visualized at 1 foot, but the patient could perceive hand motions. Patients with ETDRS visual acuities less than 5/200 were checked for the ability to perceive hand motions. Patients who could only perceive light or not perceive light were recorded as “light perception” or “no light perception”, respectively [6,7,8,9,10].

The patients who contacted the study were all provided the same introductory material by email. Patient questions were answered. No attempt was made to preselect, encourage or discourage patient application. For those patients who forwarded a suitable exam for evaluation, all patients meeting inclusion criteria and avoiding exclusion criteria were offered the opportunity to participate. This report is the result of the patients who entered the study and provided the post-operative data requested over the year following treatment. 

Patients were selected for each arm based on the degree of visual loss at the preoperative visit. Worse vision was more typical to engender an offer of Arm 2 than Arm 1. However, if there were factors that would potentially create a greater risk of complications in the judgment of the principal investigator, a less aggressive arm would be selected. After analysis, no statistical difference in response was noted between Arm 1 and Arm 2. 

All patients received anesthesia for the procedure. This was either general anesthesia under laryngeal mask anesthesia (LMA) or intravenous sedation and monitoring called monitored anesthesia care (MAC) at the discretion of the anesthesiologist based on medical assessment. 

A standard bone marrow trocar was used for bone marrow aspiration. Separation of the stem cell fraction was performed with an Arthrex cPRP and bone marrow processing system. No media or storage of cells was used. Retrobulbar and sub-,tenons injections were performed in the standard fashion. 

Preoperative examinations and follow up criteria have been presented in numerous previously published papers by the authors. They are reiterated here to facilitate understanding of the SCOTS procedures by readers: 

Each participating patient underwent an extensive discussion in which the experimental nature of the proposed surgery was stressed. All surgeries were performed in an out-patient ambulatory surgery center by one of the authors (JNW).

Examinations were performed the day before surgery and after surgery at 1 and 3 days by one of the authors (JNW). As the patients came from distant geographical locations, postoperative examinations at 1, 3, 6, and 12 months were performed by the patient’s local eye physician. Patients were included in this report if they or their eye physician had provided follow up eye exams with visual acuity testing, which included at least the 6 months post-treatment exam.

Binocular visual acuity using Snellen line equivalents of LogMAR vision was used to assess overall patient results. Individual eyes were evaluated in the same fashion. Eyes with HM or CF vision were converted to Snellen lines of vision equivalents. Per this formula, HM is considered 20/20,000, decimal 0.001 and LogMAR 3.0; CF at 2 feet is considered 20/2000, decimal 0.01, LogMAR 2.0. This conversion is derived from the LogMAR scale, which is the log of the minimum angular resolution. Individual eyes were assessed using Snellen acuity including converting CF and HM to Snellen equivalents, which ranged from 20/800 to 20/1600. Patients with a visual acuity of less than CF at 4 feet or with reports of vision from follow up exams that reported CF without a specific distance, were classified as CF vision of 2 feet and considered a Snellen acuity of 20/2000, decimal 0.01, LogMAR 2.0. All visions of CF at 4 feet or better were reported as Snellen equivalents with concomitant decimal and LogMAR visions including extrapolation of LogMAR for CF 6 feet and CF 4 feet. LP vision was not reported as LogMAR. For calculations of lines of vision and percentage improvements or loss, additional individual letters (the + or − recorded in acuity) were not utilized. In calculating the percentage of change in improved eyes, the delta or difference between the LogMAR pre-procedure acuity and post-procedure acuity was divided by the pre-procedure acuity to compare the amount of improvement to baseline vision. This can be written as pre-LogMAR – post-LogMAR/pre LogMAR.

The study was conducted in accordance with the precepts established by the Helsinki Declaration. Human investigations were performed with informed consent under the approval of an Institutional Review Board. Ethical approval code: ICMS-2013-0019, Date of approval: 30 July 2013.

## 3. Results

A summary of the patient’s demographic data and acuity results is presented in Table 1. In this study, if both eyes meet study eligibility, both eyes received stem cell treatment. Bilateral treatment was felt to be safe as the initial 75 SCOTS patients received uniocular treatment without complication.

The preoperative visual acuity ranged from counting fingers at 2 feet to 20/50. Postoperatively, the visual acuity was counting fingers at 4 feet to 20/40.

Eight patients were Arm 1 in one eye, and Arm 2 in the other eye, 5 patients were Arm 2 in each eye, one patient was Arm 3 in one eye and Arm 2 in the other, one patient Arm 3 in one eye and Arm 1 in the other, and one patient was Arm 1 in each eye. There were no intraoperative complications. 

Of the 32 treated eyes, the visual acuity of 11 (34%) experienced a 2 to 9 line improvement, 9 (28%) experienced a less than 2 line improvement, 11 (34%) remained stable, and 1 eye (3%) worsened. Improvement was experienced in 20/32 (63%) in a progressive condition. 

Analyzing binocular improvement in the 16 patients revealed that 11 (69%) experienced an improvement in their day-to-day functional vision, there was no change in 4 (25%) patients, and 1 (0.06%) patient worsened (Table 2).

Following treatment, 20 of 32 (63%) eyes experienced an improvement in visual acuity averaging 27.6% on LogMAR and ranging from 2.5% to 44.6%. The mean improvement in LogMAR was 0.963 with standard deviation (SD) of 0.42.

Analyzing acuity change in relation to treatment arm demonstrated an average improvement for 11 eyes treated in Arm 1 of 1.68 lines; for 19 eyes treated in Arm 2 of 1.29 lines; for 2 eyes treated in Arm 3 of 3.5 lines. The number of patients in Arm 3 was insufficient for comparison between arms. Assuming equal variance in an unpaired, two-tailed t-test, no statistically significant difference in visual improvement was detected in the comparison of Arm 1 with Arm 2. Assuming unequal variance (Welch t-test) and a one-tailed, paired (dependent) t-test also failed to establish a significant difference. 

For statistical evaluation, a two-sample t-test was utilized. As data was of the visual acuity of the identical eyes compared prior and post-treatment, a dependent or paired t-test was utilized. Pre-treatment, the variance was 0.2108 and post-treatment the variance was 0.1786. Because this was nearly equal, a two-tailed test was employed. In achieving a probability of less than 0.001, a t value of 4.2036 exceeded the critical value of 3.633. This demonstrated strong statistical significance. When a Welch t-test was checked to allow any difference in variance, the values were identical. 

One patient treated with Arm 2 in both eyes (OU) experienced a retinal detachment four months postoperatively, which was initially successfully repaired with a small improvement in vision, as compared to the preoperative SCOTS vision. Six months later, the patient re-detached, surgery was performed, and the retina re-attached. The patient’s final visual acuity at 18 months postoperatively improved slightly from the preoperative Count Fingers (CF) at six inches to CF one foot OD postoperatively, and more significantly, from 20/400 to 20/150 OS. One Arm 2 patient experienced a postoperative worsening of vision from 20/200 to 20/400 at nine months postoperatively. The decrease in visual acuity was felt to be a progression of the patient’s natural condition and not a complication of the SCOTS procedure.

Postoperative fundus photographs and postoperative ocular coherence tomographs (OCT) were provided variably by the patient’s local ophthalmologist in follow up exams. An analysis of the preoperative and available postoperative fundus photographs and OCT taken at the macula did not demonstrate any grossly observable differences. This may imply that the visual improvements were a result of paracrine effects on the RPE as suggested in Jiang’s laser injury work and not engraftment. However, preclinical evidence by Harris on CD 133 suggests the transdifferentiation of BMSCs to RPE cells is possible. 

Table 3 shows samples taken from the bone marrow aspirate before, and again after the centrifugation process. The first column shows the pre- and post-centrifuge cells in the five patients who had no change or worsened (one eye). The second column is of the 11 patients who had improved vision. There is a large standard deviation in each of the various groups and no statistical differences were noted in the number of cells provided between patients who improved and patients who did not.

Whether a posterior vitreous detachment (PVD) was present or did not make any difference. In comparing the different procedures, there did not appear to be a difference in the effectiveness.

If the patient presented with a preoperative visual acuity of 20/400 or less, the maximum postoperative improvement in visual acuity was 20/300. This also indicates that macular function is not restored, but there is presumably an improvement in function at the margins of the central scotoma. If the patient presented with a visual acuity of 20/200, improvement to 20/80 was seen. This implies that the penumbra of the atrophic areas still possesses macular function. A presenting visual acuity of 20/100 indicates macular function and postoperative improvement to 20/70 was observed. Early intervention is beneficial.

## 4. Discussion

Age-related macular degeneration (AMD) [11] is the main reason why older patients lose vision. It is anticipated to afflict about one hundred ninety six million individuals this year (2020) [12]. The classic appearance of AMD includes yellow-white deposits called drusen, which are composed of lipids, proteins, lipofuscin granules and small RNAs [13]. The more advanced stages of the disease may be divided into the more common “dry” or atrophic type, also called geographic atrophy, and a “wet” or neovascular type [12,13]. At the present time, there are treatments for wet AMD in the form of inhibitors of vascular endothelial growth factor, as well as older treatments such as laser photocoagulation and photodynamic therapy for patients. Certain vitamins and the eating of vegetables may extend the time before neovascularization develops for certain stages of dry AMD. However, there is no effective means to reverse or improve vision for the dry type of AMD.

The rate of progression of AMD is thought to be increased by cigarette smoking, eating dietary fats and obesity [14]. Initially, dietary supplements such as lutein, zeaxanthin and omega type three fatty acids were shown to slow AMD progression but further analysis did not prove efficacy [15].

Visual cycle modulators [16], inflammatory modulators [17] and neuroprotective agents [18] have all been studied in an attempt to slow the progression of dry AMD. Mitochondrial aging and the production of oxidative molecules are postulated to promote the initiation and advancement of AMD but no effective pharmacological interventions have proven successful.

Fifty percent of the risk of developing AMD has been explained by a complex association of genetics, environment and lifestyle. Genetic linkage analysis has identified multiple sets of genetic variants that have roles in the immune response, inflammatory processes and retinal homeostasis [19].

Regrowth of the layer of retinal pigment epithelium (RPE) using cell-based therapies has been performed but this technique relies upon the presence of remaining photoreceptors. Treating significant geographic atrophy, with the loss of all the retinal layers, remains problematic. Difficulties include the formation of functional synapses and the successful orientation and polarization of the donor photoreceptors following transplantation.

We have previously shown that the SCOTS treatment of patients with retinal and optic nerve disease including Lebers hereditary optic neuropathy (LHON) could provide visual acuity benefit, which was stable over multiple months. This included acuity increases from counting fingers to 20/100 and hand motion to 20/200 with benefit to peripheral vision and the thickness of the nerve fiber layer (NFL) of the head of the optic nerve [20]. 

We also reported regarding a larger group of patients with retinitis pigmentosa, a genetic, inherited retinopathy. The treatment in SCOTS is the first and only approach to show statistically significant visual acuity improvement (*p* < 0.016) in this hereditary, blinding condition. Following treatment, 64.7% of patients showed an average of 10.23 Snellen lines of improvement in binocular vision. Of eyes treated, 45.5% showed improvement averaging a 40.9% increase on the LogMAR vision scale with 45.5% remaining secure during the period of follow up. This is the first time any treatment has proven beneficial to acuity or stabilized vision in a hereditary retinopathy [21].

Limitations are present in any study. The patients in SCOTS often were traveling some distance and they were followed by their local ophthalmologists after the procedure. This was a positive in that the independent eye exams were thought to help eliminate any potential bias. One study limitation was that local eye doctors did not typically have ETDRS charts available to test the patients; rather Snellen acuities were provided. This excluded the potential of comparing preoperative ETDRS visions with postoperative ETDRS. Unfortunately, technicians unfamiliar with low vision patients may not allow adequate time for the patient to use eccentric fixation and therefore lower visual acuities may be recorded. In addition, despite instructions to the contrary, in many cases, postoperative visual field testing was not provided by the patient’s eye doctor, despite the ability of the patient to do so.

In 2010, Weiss performed the first subretinal surgery for a patient with AMD utilizing bone marrow-derived stem cells taken from the patient. Schwartz subsequently reported the subretinal injection of human embryonic stem cells (hESC) that had been differentiated into RPE cells. In 18 eyes treated, vision increased in 10 eyes, increased or remained stable in 7 eyes and was reduced in 1 eye. They reported complications associated with the performance of their surgery and also as a consequence of required immunosuppression. Although visual acuity improvements were marginal and immunosuppression problematic, the procedure appeared safe [22]. Clinical trials of induced pluripotent stem cells are presently underway [23].

Similarly, Song reported results of subretinal injections of hESC for AMD and Stargardts in four patients, which required the use of immunosuppression. They reported that there were no serious safety issues although one patient developed pneumonia, potentially related to immunosuppression, that was successfully treated. Lines of visual improvement were noted in three of the four eyes treated [24]. Of interest to readers may be other allogenic stem cell clinical trials including the ACT trials, Masayo Takahashi hiPSC-RPE trial, JCyte Trial and ReNeuron trial. An important advantage of autologous BMSCs is that no immunosuppression is required, avoiding the expense and risk associated with modifying the immune system.

Treatment with BMSCs has shown visual benefit for a number of ophthalmologic diseases as already outlined. The retina is a complex multilayer and multicellular structure that includes retinal pigment epithelial tissue, specialized neurons that form photoreceptors, several types of special neurons for the integration of neuronal information and the Muller cells, which may function in retinal regeneration. The improvements that have been demonstrated following treatment with BMSC in dry AMD may result from a similarly complex mix of actions on the various tissues.

A general mechanism by which BMSCs affect retinal cells is through the release of exosomes and microsomes that contain various proteins and nucleic acids. These may provide neuroprotection by increasing the resiliency and improving the function of retinal cells. Various neuroprotective substances including nerve growth factor (NGF), brain-derived neurotrophic factor (BDNF), ciliary body derived neurotrophic factor, and glial cell-derived neurotrophic factor all have been documented in exosomes. The release of micro-interference RNA (miRNA) can up- and down-regulate gene expression to the benefit of cells. Potentially, this could improve trans-retinol recovery to cis-retinal and reduce lipofuscin and drusen formation. Transfer of cytoplasmic structures via cytoplasmic bridges to adjacent cells, specifically mitochondria but potentially microtubules, has been demonstrated, which may neuroprotect and improve cell functioning.

In animal models, Muller cells will spontaneously form new retinal cells including photoreceptors, but this previously had remained in question for the human retina. However, formation of new neurons, called neuronal transdifferentiation, has now been shown to occur in the human retina following the fusion of BMSCs with Muller Cells [5]. Placement of BMSCs as provided in the SCOTS study may allow this to occur more frequently, with potential differentiation into photoreceptors a possibility. Immune modulation and reduction of inflammation that is caused by deposition of retinol dimers called A2E in retinal pigment epithelial (RPE) cells may be important.

The absence of grossly different postoperative fundus photographs or OCT suggests that the visual improvements were a result of paracrine effects on RPE as suggested in Jiang’s laser injury work and not engraftment. However preclinical evidence in Harris on CD 133 suggests transdifferentiation of BMSCs to RPE cells is possible. Greater scrutiny of cellular presence following clinical improvements may help to resolve when engraftment versus paracrine effects may predominate.

## 5. Conclusions

Dry age-related macular degeneration (AMD) is a leading cause of vision loss in the older population. The progressive decrease in central vision is highly impactful to patients with the loss of ability to drive, read unaided and see faces. There is no approved drug treatment to mitigate the natural course of visual loss associated with dry AMD. In our paper, 32 eyes with dry AMD were treated with autologous bone marrow-derived stem cells (BMSCs). Following treatment, 63% of eyes were found to have improved visual acuity averaging 27.6% on LogMAR and ranging from 2.5% to 44.6%. The visual acuity maintained stability in 34% of eyes treated over a one year follow up period. No immunosuppression was needed to support the transferred cells. Results were statistically significant (*p* < 0.001). Our evaluation supports that this approach may improve or stabilize visual acuity in the dry form of AMD. Further clinical evaluation of this treatment option is warranted.

## Figures and Tables

**Table 1:** Visual acuity results.

Patient No.	Age (yrs)/Gender	Medical History	Ocular History	Family History	PVD OD/OD	Arm #	Pre-Va OD LM (LogMAR)	Post-Va OD LM Δ LM/PreOp LM = %	Pre-Va OS LM	Post-Va OS LM Δ LM/PreOp LM = %	Comments
1	78/M	-	S/P cataract surgery OD	AMD	Y/Y	2/1	20/400 1.3	20/400 1.3	20/400 1.3	20/400 1.3	Two alcoholic drinks/day for “many years”
2	73/M	Hypertension	Laser 2006 OS CNV	-	N/N	2/1	20/200 1.0	20/100 0.7 Δ0.3/1.0 =30%	20/400 1.3	20/400 1.3	Two alcoholic drinks/day for “many years”
3	83/F	Hypertension/Hyperchole-sterolemia	S/P cataract surgery OU	-	N/N	2/1	20/400 1.3	20/400 1.3	20/200 1.0	20/80 0.6 Δ0.4/1 = 20.3%	
4	74/M	Hypertension	-	AMD	Y/Y	2/1	20/100 0.7	20/70 0.55 Δ0.15/0.7 = 21.4%	20/100 0.7	20/70 0.55 Δ0.15/0.7 = 21.4%	Two alcoholic drinks/day for “many years”
5	70/F	Cardiac Dis./Hypothyroidism	S/P cataract surgery OU/S/P vitrectomy (VH)	Cardiac Dis./DM	Y/hx vitrectomy	1/2	CF 6’ 1.85	20/400 1.3 Δ0.55/1.85 = 29.7%	CF 2’ 2.0 (20/2000)	CF 4’ 1.95 (20/1000) 1.7 Δ0.05/2 = 2.5% (20/1000) 1.7 Δ0.3/2.0 = 15%	Smoked 2–3 cigars/day for “many years”
6	74/M	Type 2 DM	S/P cataract surgery OU	DM	Y/Y	2/1	20/400 1.3	20/200 1.0 Δ0.3/1.3 = 23%	20/70 0.55	20/60 0.48 Δ0.07/0.55 = 12.7%	
7	84/F	Hypertension	S/P cataract surgery OU	AMD/glaucoma	Y/Y	1/1	CF 5’ 1.9	20/300 1.16 Δ0.74/1.9 = 38.9%	20/200 1.0	20/100 0.7 Δ0.3/1.0 = 30%	
8	77/F	Hyperthyroidism, Hypertension	S/P cataract surgery OS, Brown’s Syndrome	DM	N/Y	2/2	20/70-2 0.56	20/40-2 0.31 Δ0.25/0.56 = 44.6%	20/80+1 0.6	20/60+2 0.45 Δ0.14/0.6 = 23.3%	
9	83/F	Hypertension	S/P cataract surgery OU	AMD	Y/N	1/2	20/40 0.3	20/40 0.3	CF 6’ 1.85	20/400 1.3 Δ0.55/1.85 = 29.7%	
10	81/M	Type 2 DM/Pacemaker/Hyperthy-roidism	Laser OD CNV/S/P cataract surgery OU/S/P VEGF injections OS/S/P Vitrectomy-membrane stripping OD	AMD/DM	N/Y	3/2	Cf 6’ 1.85	20/400 1.3 Δ0.55/1.85 = 29.7%	20/50 0.4	20/40 0.3 Δ0.1/0.4 = 25%	
11	78/F	Hypertension	S/P cataract surgery OU/glaucoma OU	AMD/glaucoma	Y/Y	2/2	20/400 1.3	20/400 1.3	20/200 1.0	20/400 1.3 Δ0.3/1.0 = (−30%)	Smoked x 30 years, dc’ed 3 yrs. ago
12	86/F	Hypertension	S/P cataract surgery OU	-	Y/Y	2/2	20/400 1.3	20/300 1.16 Δ0.14/1.3 = 10.8%	20/400 1.3	20/350 1.23 Δ0.07/1.3 = 5.4%	4M postop, RD OS, successfully repaired
13	78/F	Hypertension	S/P cataract surgery OU	-	Y/Y	2/2	20/400 1.3	20/400 1.3	20/400 1.3	20/400 1.3	
14	70/F	Hypertension	-	AMD	Y/Y	2/2	20/200+1 1.0	20/100 0.7 Δ0.3/1.0 = 30%	20/200+1 1.0	20/100 0.7 Δ0.3/1 = 30%	
15	69/M	Hypertension/Pacemaker	“Many” VEGFI injections OU x yrs	DM	Y/Y	2/1	20/400 1.3	20/400 1.3	20/100+1 0.7	20/80 0.6 Δ0.1/0.7 = 14.3%	Geographic atrophy without scarring
16	90/M	Hypertension/S/P CABG	“Many” VEGFI injections OU x yrs/S/P laser OD/cataract surgery OU	AMD	Y/Y	1/3	20/50-2 0.45	20/60+1 0.46 Δ0.01/0.45 = (−2.2%)	20/400 1.3	20/400 1.3	
							18.71/16 = 1.169	15.44/16 = 0.965	17.3/16 = 1.081	15.36/16 = 0.96	
							total pre 36.01/32	= 1.1253 pre LM	total post 30.8/32	= 0.9625 post LM	delta’ = 0.1628
							used CF			used CF	
							delta/pre for all	14.50%			
							Improve OD 3.28/9	ImproveOS 2.23/11	5.51/20 = 27.6%		

**Table 2:** Change in snellen lines of vision.

Patient #	Postop Va Change OD	Postop Va Change OS	Postop Total Change in Best Va OU
1	No Change	No Change	No Change
2	+3 lines	No Change	+3 lines
3	No Change	+4 lines	+4 lines
4	+1.5 lines	+1.5 lines	+1.5 lines
5	+3 lines	CF2’ to CF4’	+3 lines
6	+3 lines	<+1 line	<+1 line
7	+6 lines	>2 lines	>2 lines
8	<+3 lines	<+1 line	<+3 lines
9	No Change	>+6 lines	No Change
10	>+7 Lines	<+1 line	<+1 line
11	No Change	−3 lines	−3 lines
12	+1 line	<+1 line	+1 line
13	No Change	No Change	No Change
14	<+3 lines	<+3 lines	<+3 lines
15	No Change	<+1 line	<+1 line
16	+/− No Change	No Change	No Change

**Table 3:** Displays the hematologic data.

		Vision Worsened or No Change				Vision Improved			
		AVG	±	SD	N	AVG	±	SD	N
PRE	TNC (total cells)	3,506,640,000	±	2,245,323,702	5	3,397,163,636	±	2,733,548,103	11
	RBC (total cells)	609,660,000,000	±	114,498,188,632	5	523,415,254,545	±	191,745,553,487	11
	Plt (total cells)	17,558,400,000	±	10,649,249,589	5	14,269,090,909	±	11,184,658,103	11
BMAC Post	TNC (total cells)	1,191,480,000	±	917,372,055	5	1,183,881,818	±	1,004,155,772	11
	RBC (total cells)	4,748,170,000	±	8,914,776,662	5	11,493,979,091	±	9,554,840,765	11
	Plt (total cells)	4,210,500,000	±	5,474,659,624	5	3,142,636,364	±	5,975,152,831	11
